# 

**Published:** 1884-01

**Authors:** 


### INDEX

TO THE

### HOMŒOPATHIC PHYSICIAN.

### VOLUME IV.

PAGE

Acon., . . . . . . . . . . . . . . . . . 66, 177
Aesc-hipp., . . . . . . . . . . . . . . . 119
Aethusa, . . . . . . . . . . . . . . . . 121
Agaricus, . . . . . . . . . . . . . . . 66, 101
Ailanthus, . . . . . . . . . . . . . . . 43
Allen, John V. Cholera Infantum, . . . 249
Althæa off., . . . . . . . . . . . . . . 156
Aloe, . . . . . . . . . . . . . . . . . 291
Alum, . . . . . . . . . . . . . . 66, 101, 120
Ambra, . . . . . . . . . . . . . . . . 66
American Institute, The. P. P. Wells, . 49
" " " F. R. McManus, 156
Amm. carb., . . . . . . . . . . 24, 101, 120
Amm. caus., . . . . . . . . . . . . . . 66
Amm. mur., . . . . . . . . . . . . 100, 120
Anacard., . . . . . . . . . . . . . . 66, 101
Anæsthetics in Labor. C. L. Swift, . . . 58
Analysis of Fincke's High Potencies by the American Institute. B. Fincke, . . . . 141
August., . . . . . . . . . . . . . . . . 66
Ant. crud., . . . . . . . . . 66, 101, 120, 253
Ant-tart, . . . . . . . . . . . . . . . . 66
Apis, . . . . . . . . . . . 120, 148, 175, 332
Asterias rub., . . . . . . . . . . . . . . 66
Arg-nit., . . . . . . . . . . . . . . . . 66
Arnica, . . . . . . . . . 63, 66, 120, 253, 254
Arsenic, . . . . 66, 120, 148, 177, 253, 254, 291
Arum tri, . . . . . . . . . . . . . . . 43
Asafœt., . . . . . . . . . . . . . . . . 66
Atkins, E. B. Medical Science, . . . . . 94
Aurum, . . . . . . . . . . . . . . . 101, 288
Aur-mur., . . . . . . . . . . . . . . . 101
Ayers on Diseases of Rectum, . . . . . . 248
Baptisa, . . . . . . . . . . . . 25, 254, 256
Baryta carb., . . . . . . . . . . . . 66, 101
Bayard, Edward. Nosodes, . . . . . . . 245
Bellad., . . . . . . . . . . . . . 27, 66, 175
Benz-acid, . . . . . . . . . . . . . . . 177
Berberis, . . . . . . . . . . . . . . . . 45
Berridge, E. W.
- Clinical Cases, . . . . . . . . . . . 45, 294
- Clinical and Pathological Notes, . . . 288
- I. H. A. and its Supposed Shortcomings, 32
- Lac. caninum, . . . . . . . . . . 103, 297
- Proving of Lac Vac. Defl. . . . . . . 74
- Peculiar Case of Nymphomania, . . . 294

PAGE

Best Colleges and Dr. McKibben. P. P. Wells, . . . . . . . . . . . . . . . . 315
Best Disinfectant, The, . . . . . . . . . 287
Bismuth, . . . . . . . . . . . . . . . . 177
Book Notices.
- Address before Homœopathic Medical Society of New York State, . . . . 195
- American Medicinal Plants. C. F. Millspaugh, . . . . . . . . . . . . 299
- Annual Address before California State Society. G. M. Pease, . . . . . . . 232
- Cholera, its Prevention and Treatment. D. N. Rây, . . . . . . . . . . . . 265
- Cholera, Treatment of. C. H. Gatchell, 333
- Diphtheria and its Management. Thos. Nichol, . . . . . . . . . . . . . 195
- Drugs and Medicines of North America. Lloyd, . . . . . . . . . . . . . . 266
- Hand-book of Skin Diseases and their Hom. Treatment. John R. Kippax. . . . . . . . . . . . . . . . 28
- Hering Memorial Volume. Drs. Raue, Knerr, and Mohr, . . . . . . . . . 196
- Obstetric Mentor. Clarence M. Conant, 76
- Physician's Special Rate Check-book. Wm. J. Guernsey, . . . . . . . . 48
- Proceedings of Homœopathic Medical Society of Ohio, . . . . . . . . . 333
- Sexual Neuresthenia. Geo. M. Beard, 196
- Surgical Emergencies. J. G. Gilchrist, 333
- Some Diseases of the Rectum. Mortimer Ayers, . . . . . . . . . . . 196
- The Knowledge of the Physician. Richard Hughes, . . . . . . . . . 299
- Transactions of Homœopathic Medical Society of Pennsylvania, . . . . . 48
- Treatise on Intra-cranial Diseases. Chas. Porter Hart, . . . . . . . . 104
- Therapeutics of Intermittent Fever. H. C. Allen, . . . . . . . . . . . . . 265
- Uterine Displacements. S. J. Donaldson, . . . . . . . . . . . . . . . 132
- Uterine Therapeutics. H. Minton, . 74
- Vaccinosis and its Cure by Thuja, etc. J. Compton Burnett, . . . . . . . 232

Borax, . . . . . . . . . . 66, 73, 120, 294, 331

PAGE

Bovista, . . . . . . . . . . . . . . . . 66
Bromium, . . . . . . . . . . . . . . . 66, 119
Bryonia, . . . 46, 67, 71, 101, 102, 120, 177, 257
Buckmann, Remarkable Cure by Phosphorus, . . . . . . . . . . . . . . . . 221
Cactus gr., . . . . . . . . . . . . . . . . 66
Calcarea, . . 66, 101, 103, 127, 158, 177, 251, 288, 289, 291
Calc. phos., . . . . . . . . . . . . . . 120, 330
Cancrum Oris-Baptisia. Walter M. James, 25
Cann-sat., . . . . . . . . . . . . . . 66
Cantharis, . . . . . . . . . . . 43, 66, 101, 120
Capsicum, . . . . . . . . . . . . . . 66, 102
Carbo-an., . . . . . . . . . . . . . 66, 101, 192
Carbo veg., . . . . . . . . . . . . . . 66, 177
Causticum, . . . . . . . . . . . . . . 66, 101
Central New York Hom. Med. Society, Proceedings of the, . . . . . . . . . 62, 155
Chamomilla, . . . . . . . . . . 61, 66, 176, 289
Chelidonium, . . . . . . . . . . . . . 71, 289
China, . . . . . . . . . . . . 66, 72, 101, 177
Chinin-sul., . . . . . . . . . . . . . . . 66
Cholera Infantum. Ad Lippe, . . . . . 172
" " John V. Allen, . . . 249
Cholera, Snake-bite, and their Lessons. P. P. Wells, . . . . . . . . . . . . . . 267
Cicatrix Removed by Medicine, A. J. T. Kent, . . . . . . . . . . . . . . . . 158
Cicuta, . . . . . . . . . . . . . . . . 101
Cina, . . . . . . . . . . . . . . . . . 66
Cinchona, . . . . . . . . . . . . . . . 48, 288
Clematis, . . . . . . . . . . . . . . . . 120
Clinical Cases.
E. W. Berridge, . . . . . . . . 45, 288, 294
Wm. Jefferson Guernsey, . . . . 194, 261
Chas. T. Harris, . . . . . . . . . . . 265
J. T. Kent, . . . . . . . . . . 127, 298, 330
A. B. Knott, . . . . . . . . . . . . . 231
C. Lippe, . . . . . . . . . . . . . . 193
Mahlon Preston, . . . . . . . . . . . 158
Julius Schmitt, . . . . . . . . . . 189, 293
J. D. Turrill, . . . . . . . . . . . . 102
Clinical and Pathological Notes. E. W. Berridge, . . . . . . . . . . . . . . . 288
Cocculus, . . . . . . . . . . . . . . . 103
Coffea, . . . . . . . . . . . . . . . . 66
Coffee: its Therapeutics, . . . . . . . . 81
Colchicum, . . . . . . . . . . . . . . 66, 258
Colocynth, . . . . . . . . . . . . 120, 178, 261
Conium, . . . . . . . . . . . . . . . 66, 120
Convallaria majalis: a Proving. Irvin J. Lane, . . . . . . . . . . . . . . . . . 122
Coral-rub., . . . . . . . . . . . . . . . 66
Creosot., . . . . . . . . . . . . . . . 66, 178
Criticism, A Sharp, of Homœopaths, etc., . 90
Criticism of Dr. Wilson's Rejoinder to Dr. Wells on the Vital Force. B. Fincke, . 77
Crocus, . . . . . . . . . . . . . . . . 66
Crotalus, . . . . . . . . . . . . 66, 274, 275
Croton-tig., . . . . . . . . . . . . . . 176
Cuprum, . . . . . . . . . . . . . . . . 66
Curantur or Curentur. R. E. Dudgeon, . 179
" " Ad. Lippe, . . . 243
Curious Statement, A, . . . . . . . . . 203
Cyclamen, . . . . . . . . . . . . . . . 120
Desires and Aversions, Supplement. Wm. Jefferson Guernsey, . . . . . . . . . 16 pp.
Diadema, . . . . . . . . . . . . . . . 66
Diarrhœa—Podophyl. C. P. Jennings, . 262
Digitalis, . . . . . . . . . . . 45, 46, 66, 179
Dioscorea, . . . . . . . . . . . . . . . 148
Diphtheria. E. B. Nash, . . . . . . . 39, 331
Diseases from the Influence of the Passions. B. W. Richardson, . . . . . . . 282
Distinctive Symptoms. T. L. Brown, . . 279

PAGE

Drosera, . . . . . . . . . . . . . . . . 66
Dudgeon, R. E.
Curantur or Curentur, . . . . . . . 179
Hahnemann's Homœopathic Formula, 317
Hahnemann and Cholera Microbes, . 335
Dulcamara, . . . . . . . . . . . . . . 66
Dysmenorrhœa. J. T. Kent, . . . . . . 330
Dysmenorrhœa and Sterility, Cured by Colocynth. Wm. Jefferson Guernsey, . 261
Ebstein, Professor. Diet for the Corpulent, 153
Eczema Cured by Vaccination, . . . . . 41
Eczema Capitis, Therapeutics of, . . . . 101
Emergencies—Euthanasia. J. T. Kent, . 321
Eucalyptus, . . . . . . . . . . . . . . 291
Eupat-perf., . . . . . . . . . . . . . 67, 258
Euphrasia, . . . . . . . . . . . . . . 66, 120
Experiments with High Potencies, etc., . 92
Fago, . . . . . . . . . . . . . . . . . 101
Farfara, Proving of. Irvin J. Lane, . . . 323
Ferrum, . . . . . . . . . . . . . 66, 120, 231
Ferrum carb., . . . . . . . . . . . . . 72
Fluoric acid, . . . . . . . . . . . . . 120
Freedom of Medical Opinion Illustrated, . 113
Fincke, B.
Analysis of Fincke's High Potencies by the Am. Institute, . . . . . . . 141
A Criticism of Dr. Wilson's Rejoinder to Dr. Wells on the Vital Force, . . 77
Potentiation a Consequence of the Law of Similia, and vice versa, from the Hahnemannian Standpoint, . . . . 301
Rapid Cure of a Disease thus far Known as Absolutely Fatal, . . . 70
Gambogia, . . . . . . . . . . . . . . . 66
Gee, W. S., A Case of Hay Fever, . . . . 355
Gelsemium, . . . . . . . . . . . . . . 256
Granatum, . . . . . . . . . . . . . . 66
Graphites, . . . . . . . . . . . . . . 66, 101
Gregg, R. R. A Medical Student's Clinical Observations in the two Schools of Medicine, . . . . . . . . . . . . . . . 206
Guare, . . . . . . . . . . . . . . . . . 102
Guernsey, Wm. Jefferson.
Desires and Aversions, Supplement, 16 pp.
A Clinical Case, . . . . . . . . . . 194
Dysmenorrhœa and Sterility Cured by Colocynth, . . . . . . . . . . . 261
What I Know About Phytolacca, . . 357
Hahnemann's Homœopathic Formula. R. E. Dudgeon, . . . . . . . . . . . . . 317
Hahnemann and Cholera Microbes. R. E. Dudgeon, . . . . . . . . . . . . . . 335
Hahnemann on Large Doses of Homœopathic Remedies, . . . . . . . . . . . 281
Hahnemann Medical Association of Louisiana, . . . . . . . . . . . . . . . . 155
Hahnemann and Ziemssen, . . . . . . . 167
Hall, John. Malarial Fever as Suppressed, with Illustrative Cases, . . . . . . . . 181
Harris, C. T. Clinical Case, . . . . . . . 264
Hay Fever, A Case of. W. S. Gee, . . . . 355
Helleborus, . . . . . . . . . . . . . 130, 179
Hepar, Sulph., Calc., . . . . . . . . 66, 101, 149
Homœopathy Among American Indians, . 42
Homœopathic Colleges. Alice B. McKibben, . . . . . . . . . . . . . . . . . 252
Homœopathic Medical Society of New York, . . . . . . . . . . . . . . . . . 91
Hura, . . . . . . . . . . . . . . . . . 101
Hydrastis, . . . . . . . . . . . . . . . 122
Hyoscyamus, . . . . . . . . . 66, 179, 254, 255
Idiosyncrasy. J. T. Kent, . . . . . . . 319
Ignatia, . . . . . . . . . . . . . . . . 66
Importance of Mental Symptoms, . . . . 25
Infallible Microscope, . . . . . . . . . 115, 167

PAGE

Ingalls, F. W. Proper Treatment of Malarial Fevers, . . . . . . . . . . 170
International Hahnemannian Association.
Bureaus for 1884, . . . . . . . . . . . . 66
Its Supposed Shortcomings E. W. Berridge, . . . . . . . . . . . . . . 35
The June Meeting. Dr. Pearson, . . . 114
Meeting for 1885, . . . . . . . . . . . 351
I nvoluntary Stools, Phosphorus. J. T. Kent, . . . . . . . . . . . . . . . . 292
Iodium, . . . . . . . . . . . . . . . . . 66, 179
Ipecacuanha, . . . . . . 66, 72, 178, 251, 258
Iris vers, . . . . . . . . . . . . . . . 177
Isopathy of the Past and the Future, . . . 23
James, Walter M. Cancrum Oris-Baptisia, 25
Gouty Rheumatism. Lachesis, . . . . 26
Jennings, C. P. Diarrhœa-podophyl., . . 262
Juglans Regia, . . . . . . . . . . . . 102
Kali bichro., . . . . . . . . . . . . . 259, 260
Notes on. J. T. Kent, . . . . . . . 150
Kali carbonicum, . . . . . . 24, 66, 101, 121
Kali chloric., . . . . . . . . . . . . . . 66
Kali iod., . . . . . . . . . . . . . . . 66
Kali sulph., . . . . . . . . . . . . . . 66
Kent, J. T.
Cicatrix removed by Medicine, . . . . 158
Case of Chronic Arthritis, . . . . . . 126
Clinical Notes, . . . . . . . . . . . 129
Clinical Cases, . . . . . . . . . . . 298
Dysmenorrhœa, . . . . . . . . . . 330
Emergencies, Euthanasia, . . . . . . 321
Idiosyncrasy, . . . . . . . . . . . 313
Involuntary Stools, Phosphor, . . . . 292
Kali bichromicum, . . . . . . . . . 150
Malarial Fevers, Therapeutics, . . . . 253
Mammary Tumor, Carbo an., . . . . 191
Recurrent Fibroid, Silicea, . . . . . 193
Resembling Acute Bright's, . . . . . 298
The Simillimum, . . . . . . . . . . . 164
Urticaria Appearing Annually, . . . 262
Knott, A. B. Clinical Case, . . . . . . . 231
Lac-caninum, . . . . . . . . . . . 64, 104, 297
Lac. vac., Defloratum, . . . . . . . . . 74
Lachesis, . 26, 65, 66, 101, 149, 190, 274, 275, 289, 295
Lane, Irwin J. Proving of Convallaria, . 122
Proving of Farfara, . . . . . . . . 323
*Lancet*, The London, on Narcotics, . . . . 274
Laurocerasus, . . . . . . . . . . . . . 121
Ledum, . . . . . . . . . . . . . 66, 149, 150
Leonard, W. E. Clinical Cases, . . . . . 26
Lippe, Adolph.
Cholera Infantum, . . . . . . . . . 172
Curantur or Curentur . . . . . . . 243
Nosebleed, with Clinical Reflections, . 65
Psorinum: Clinical Reflections, . . . 83
Dr. Pearson and the I. H. A., . . . . 18
Recognition, . . . . . . . . . . . . 32
Relation of Homœopathy to Hahnemann, . . . . . . . . . . . . . . 115
The Sifting of our Materia Medica, . 311
Lippe, C. A Clinical Case, . . . . . . . . 193
Lobelia, . . . . . . . . . . . . . . . . 119
Lycopodium, . 26, 47, 66, 101, 128, 289, 291, 332
Lycopus, . . . . . . . . . . . . . . . 257
Mac Manus, F. R. The American Institute . . . . . . . . . . . . . . . . 156
Magn-carb., . . . . . . . . . . . . . 66, 101
Magn-mur., . . . . . . . . . . . 66, 101, 121
Magn-sul., . . . . . . . . . . . . . 66, 102
Malarial Fever, the Proper Treatment of. F. W. Ingalls, . . . . . . . . . . . . 170
Malarial Fever, Therapeutics. J. T. Kent, 254
Malarial Fever, as Suppressed, with Illustrative Cases. John Hall, . . . . . . . 181

PAGE

Mammary Tumor, Carbo an. J. T. Kent, . 191
Materia Medica—A Science, Its Nature, Uses, and How it is to be Used. P. P. Wells, . . . . . . . . . . . . . . . . 233
Medical Education and our Colleges as Contributors Thereto. P. P. Wells, . . . 197
Medical Science. E. B. Atkins, . . . . . 94
Medical Student's Clinical Observation in the Two Schools of Medicine, A. R. R. Gregg, . . . . . . . . . . . . . . . 206
Medorrhinum, . . . . . . . . . . . . . 289
Menyanthes, . . . . . . . . . . . . . . 66
Mephites, . . . . . . . . . . . . 66, 121, 288
Mercurius, . . . . . . . . . . . 66, 101, 132
Merc-corr., . . . . . . . . . . . . . . . 66
Merc-cyan., . . . . . . . . . . . . . . 66, 71
Merc-iod., . . . . . . . . . . . 101, 121, 130
Mercurialis, . . . . . . . . . . . . . . 66
Mezereum, . . . . . . . . . . . . . 101, 121
Millefolium, . . . . . . . . . . . . . . 66
Mistaken Sex, A Case of, . . . . . . . . 87
Moschus, . . . . . . . . . . . . . . 66, 101
Mur. acid, . . . . . . . . . . . . 66, 101, 256
Myrica cirif, . . . . . . . . . . . . . . 159
Nash, E B. Diphtheria, . . . . . . . . 39, 331
Report on Lac caninum, . . . . . . 64
Natr-carb., . . . . . . . . . . . . . 66, 109
Natr-mur., . . . . . . . 66, 101, 121, 178, 186
Natr-sul., . . . . . . . . . . . . . . . 66
Nitr-acid, . . . . . . . . . . 66, 101, 178, 288
Nitrum, . . . . . . . . . . . . . . . . 121
Nosebleed; with Clinical Reflections. Ad. Lippe, . . . . . . . . . . . . . . . . 65
Nosodes. Edward Bayard, . . . . . . . 245
Notes and Notices, . . 28, 48, 168, 299, 334, 362
Nux-mos., . . . . . . . . . . . . . . . 121
Nux-vomica, . . . . . . . . . . . . . 36, 148
Observations on Homœopathy. Dr. Gross, . 99
Opium, . . . . . . . . . . . . . . . . 179
Oreodaphne, . . . . . . . . . . . . . . 69
Paris quad., . . . . . . . . . . . . . . 66
Overfeeding and Summer Complaint, . . . 151
Pathology Applied. Julius Schmitt, . . . 189
Paullin, . . . . . . . . . . . . . . . . 178
Pearson, C. June Meeting of the I. H. A., 114
Pearson, Dr., and the I. H. A. Ad. Lippe, 18
Peculiar Antipathies, . . . . . . . . . . 188
Peculiar Case of Nymphomania A. E. W. Berridge, . . . . . . . . . . . . . . 294
Petroleum, . . . . . . . . . . 45, 66, 101, 178
Phosphorus, . . 66, 101, 121, 178, 223, 289, 291, 292, 297
Phosp-acid, . . . . . . . . . . . . . . 66
Phytolacca, . . . . . . . . . . . . 121, 359
What I Know of Phytolacca. Wm. Jefferson Guernsey, . . . . . . . 357
Placenta, Let Nature Remove the, . . . 122
Platinum, . . . . . . . . . . . . . . . 61
Podophyllum, . . . . . . . . . 121, 176, 262
Point in Prognosis, A, . . . . . . . . . 88
Preston, Mahlon. A Clinical Contribution, . . . . . . . . . . . . . . . . 158
Proving of Lager Beer. S. Swan, . . . . 88
Psorinum: Clinical Reflections. Ad. Lippe, 83
Psorinum, . . . . . . . . . . 84, 85, 101, 121
Pulsatilla, . . . . . . 66, 72, 121, 288, 291, 292
Quiet as a Factor in the Treatment of Children. Anna C. Howland, . . . . . 169
Ratanhia, . . . . . . . . . . . . . . . 66
Reason of the Faith that is in Me. S. Swan, . . . . . . . . . . . . . . . . 55
Recognition. Ad. Lippe, . . . . . . . . 32
Recurrent Fibroid Cured by Silicea. J. T. Kent, . . . . . . . . . . . . . . . . 193
Rejected Cases. E. W. Berridge, . . . . 259

PAGE

Relation of Homœopathy to Hahnemann. Ad. Lippe, . . . . . . . . . . . . . . 115
Rhododendron, . . . . . . . . . . . . 66, 101
Rhus-rad., . . . . . . . . . . . . . . . 263
Rhus-tox., . . . . . . . . . . . 66, 129, 257
Ruta, . . . . . . . . . . . . . . . 66, 101
Sabadilla, . . . . . . . . . . . 66, 101, 121
Sabina, . . . . . . . . . . . . . . . . 66
Sarsaparilla, . . . . . . . . . . . . . 66, 178
Schmitt, Julius.
  Cases from My Note-Book, . . . . . . 293
  Pathology Applied, . . . . . . . . . 189
Secale corn., . . . . . . . . . . . . . . 66
Selenium, . . . . . . . . . . . . . . . 101
Senega, . . . . . . . . . . . . . . . . 66
Sensible Advice, . . . . . . . . . . . . 187
Sepia, . . . . . . . . . . . . 66, 132, 149, 288
Sifting of Our Materia Medica. Ad. Lippe, 311
Silicea, . . . . . . . . 66, 101, 103, 149, 178, 193
Simillimum, The. J. T. Kent, . . . . . 164
Skinner's, Thos. F. C., High Potencies, . 82
Smith, C. Carleton.
  Amm. carb. and Kali carb. in Morning Cough, . . . . . . . . . . . . . 24
  Arum tri. and Ailanthus Compared, . 43
  Whitlow, . . . . . . . . . . . . . 141
Some Good Books, . . . . . . . . . . . 38
Spigelia, . . . . . . . . . . . . . . . 119
Spongia, . . . . . . . . . . . . . . . . 66
Stannum, . . . . . . . . . . . . . . 66, 67
Staphisagria, . . . . . . . . . . . . . 101
Strontiana carb., . . . . . . . . . . . . 66
Sulphur, . . . . 66, 101, 122, 127, 176, 289, 291
Sulph-acid, . . . . . . . . . . . . . 66, 178
Swan, S.
  Proving of Lager Beer, . . . . . . . 88
  The Reason for the Faith that is in Me, 55
  Notation, . . . . . . . . . . . . . 141
Swift, C. L. Anæsthetics in Labor, . . . 58
Symptom Covering, . . . . . . . . . . 9

PAGE

Syphilinum, . . . . . . . . . . . . . . 293
Tabacum, . . . . . . . . . . . . . . 122, 290
Taraxacum, . . . . . . . . . . . . . . . 67
Tarentula, . . . . . . . . . . . . . . . 122
Terebinthina, . . . . . . . . . . . . . . 66
Thea, . . . . . . . . . . . . . . . . . 291
Theridion, . . . . . . . . . . . . . . . 101
Thuja, . . . . . . . . . . . . . . . . . 66
Turrill, J. D. Clinical Cases, . . . . . . 112
Vaccination, . . . . . . . . . . . . . 41, 291
Verbascum, . . . . . . . . . . . . . . . 46
Veratrum-alb., . . . . . . . . . 47, 66, 101, 178
Vinca, . . . . . . . . . . . . . . . . . 66
Viola od., . . . . . . . . . . . . . . . 66
Viola tr., . . . . . . . . . . . . . . . 101
Vital Force. P. P. Wells, . . . . . . . 11
Vital Force and *The Advance*, . . . . . . 161
Vomiting of Milk, . . . . . . . . . . . 83
Washing, . . . . . . . . . . . . . . . 119
Wells, P. P.
  "Best Colleges" and Dr. McKibben, . 315
  Cholera, Snake-bite, and their Lessons, 267
  Materia Medica—A Science, Its Nature, Uses, and How it is to be Used, . . 233
  Medical Education and Our Colleges as Contributors thereto, . . . . . . 197
  Review of Report of Chairman of the Bureau of Materia Medica of the Institute, . . . . . . . . . . . . 105
  The American Institute, . . . . . . 49
  Then and Now, . . . . . . . . . . . 339
  Vital Force, . . . . . . . . . . . . 11
  Vital Force and *The Advance*, . . . . 161
  What is Homœopathic Prescribing? . 133
  What Shall we Treat? . . . . . . . 29
What I Know about Phytolacca. Wm. Jefferson Guernsey, . . . . . . . . . 357
Whitlow. C. Carleton Smith, . . . . . . 147
Zincum, . . . . . . . . . . . . . . . . . 179

VOL. IV. JANUARY, 1884. No. 1.

### THE HOMŒOPATHIC PHYSICIAN,

A MONTHLY JOURNAL OF MEDICAL SCIENCE.

"*Seek the Truth: come whence it may, cost what it will.*"

E. J. LEE, M. D.,
EDITOR.

CONTENTS:

(See inside of Cover.)

NOTICE.

***All articles for publication, books for review, business communications, etc., must be sent to Editor, 2109 Chestnut Street, Philadelphia.***

